# Nifty fifty

**DOI:** 10.5051/jpis.2010.40.3.103

**Published:** 2010-06-25

**Authors:** Tae-Il Kim

I could not but love New Zealand when I was a special forces army officer, because I came to realize that its 6,000 soldiers willingly entered the Korean War to help my country to maintain its freedom. When I was honorably discharged from the army service and attended a scientific conference in Rotorua as a faculty member, I once more fell in love with New Zealand because of its natural beauty.

As a matter of fact, the quintessential reason for my New Zealand fever is Dame Kiri Te Kanawa, who is the most famous NZ-born soprano and opera diva in the world today. On December 2007, I was captured by her elegant presence and celestial voice on the Hong Kong Cultural Centre Concert Hall stage. When she was performing her 50th birthday concert in the splendor of London's Royal Albert Hall in 1994, this much-loved diva replied to her fans who were celebrating her birthday as follows, "Singing is the great joy of my life. Thank you because fifty is nifty!"

Her briskness prevents us from being overcome with the nameless fear of getting old. It is interesting that emotional experience improves with age and the old are good at controlling their emotions and experience negative affects less frequently compared to the young.

Confucius, who was a famous Chinese philosopher, regarded the age as a measure for human maturity. The age of thirty is taken to be the age of establishing oneself. The age of forty is considered to be the age free from vacillation. The age of fifty is looked upon as the age of achieving reason from above.

Korean Academy of Periodontology (KAP), which issued its first official Journal of Korean Academy of Periodontology (JKAP) in 1971, is reaching its 50th year of existence this year. KAP established its journal as a credible source of periodontal science at the "age of thirty" and was not biased in either the field of periodontology or implantology at the "age of forty." When we were on the brink of the "age of fifty," we fortunately realized the necessity for international communication and successfully transformed the JKAP into the Journal of Periodontal & Implant Science (JPIS). This proceeded favorably and was fully covered by KoreaMed, Synapse, KoMCI, CrossRef and Google Scholar. Recently, we have a special reason for congratulation. JPIS is now proud to participate in PubMed and PubMed Central beginnig this May.

I would recommend that you enjoy looking at 5 original articles and 2 case reports within this issue, but you should not forget to give KAP hearty cheers, "Thank god because fifty is nifty!"

